# Targeting and Insertion of Membrane Proteins in Mitochondria

**DOI:** 10.3389/fcell.2021.803205

**Published:** 2021-12-24

**Authors:** Ross Eaglesfield, Kostas Tokatlidis

**Affiliations:** Institute of Molecular Cell and Systems Biology, College of Medical, Veterinary and Life Sciences, University of Glasgow, University Avenue, Scotland, United Kingdom

**Keywords:** mitochondria, membrane proteins, assembly, mitochondrial chaperones, translocons

## Abstract

Mitochondrial membrane proteins play an essential role in all major mitochondrial functions. The respiratory complexes of the inner membrane are key for the generation of energy. The carrier proteins for the influx/efflux of essential metabolites to/from the matrix. Many other inner membrane proteins play critical roles in the import and processing of nuclear encoded proteins (∼99% of all mitochondrial proteins). The outer membrane provides another lipidic barrier to nuclear-encoded protein translocation and is home to many proteins involved in the import process, maintenance of ionic balance, as well as the assembly of outer membrane components. While many aspects of the import and assembly pathways of mitochondrial membrane proteins have been elucidated, many open questions remain, especially surrounding the assembly of the respiratory complexes where certain highly hydrophobic subunits are encoded by the mitochondrial DNA and synthesised and inserted into the membrane from the matrix side. This review will examine the various assembly pathways for inner and outer mitochondrial membrane proteins while discussing the most recent structural and biochemical data examining the biogenesis process.

## Introduction

Mitochondria are critically important for metabolism and a whole range of cellular functions, while they also play an essential role in programmed cell death. The mitochondrial proteome is made up of around 1,500 different proteins in humans and around 1,000 proteins in simpler eukaryotic organisms like *Saccharomyces cerevisiae* (Rath et al., 2021; Song et al., 2021). From these proteins, only 13 are encoded by mitochondrial DNA (mtDNA) in humans and 8 in *S. cerevisiae* (7 of which encode subunits of the oxidative phosphorylation complexes). Therefore, the majority of the mitochondrial proteins (about 99% of them) are nuclear-encoded, synthesised in the cytosol and then imported into their correct location within the organelle. The protein import system is very elaborate and depends on multiprotein complexes called translocons that reside in each one of the mitochondrial sub-compartments (Schmidt et al., 2010; Pfanner et al., 2019). The main pathway for mitochondrial proteins is the presequence pathway that guides soluble proteins into the mitochondrial matrix and accounts for almost two thirds of all mitochondrial protein import. On the other hand, the mitochondrial membrane proteins that reside in the inner or the outer membranes follow their own dedicated import routes that not only target the proteins to the correct mitochondrial membrane but also specifically insert them stably within the lipid bilayer (Wiedemann and Pfanner, 2017).

**FIGURE 1:**
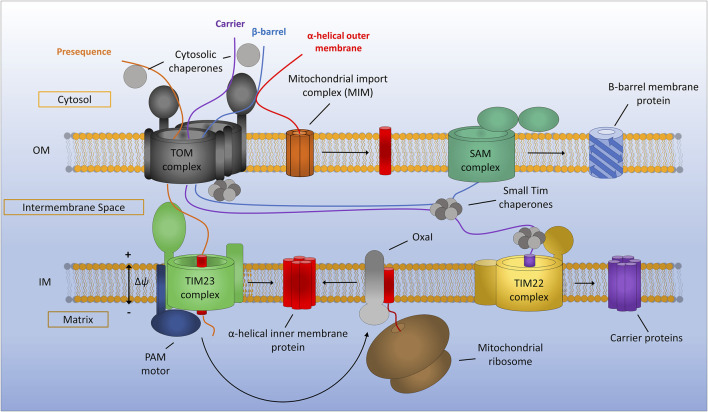
General assembly pathways for mitochondrial membrane proteins. Presequence containing proteins are recognised by the translocon of the outer membrane (TOM) complex and translocated across the outer membrane before being assembled *via* the translocon of the inner membrane (TIM23) complex. TIM23 requires a potential gradient across the inner membrane and can either laterally diffuse stop-transfer hydrophobic *α*-helices directly into the inner membrane or translocate proteins all the way to the matrix *via* the PAM motor, which can be subsequently inserted into the inner membrane by the Oxa1 insertase. Mitochondrial carrier proteins are maintained in an import competent state by cytosolic chaperones before being translocated across the outer membrane by the TOM complex. Once in the IMS carrier precursors are chaperoned by the small TIMs to the TIM22 complex where insertion into the inner membrane occurs. Beta-barrel proteins of the outer membrane are first translocated into the IMS *via* the TOM complex. The small TIM chaperones then transfer *β*-barrel precursors from the TOM to the sorting and assembly machinery (SAM) complex where insertion and assembly of the *β*-barrels takes place. Alpha-helical outer membrane proteins are most often inserted into the membrane directly from the cytosol *via* the mitochondrial import machinery (MIM) complex.

The mitochondrial *β*-barrel membrane proteins are found only in the outer membrane (OM), whilst *α*-helical membrane proteins (with either a single or multiple transmembrane domains) are present in both the outer (OM) and inner (IM) mitochondrial membranes (Figure 1). Insertion of all *β*-barrel proteins into the OM is thought to occur post-translationally (Lee et al., 2014), whilst insertion of the very few, highly hydrophobic IM proteins that are encoded by the mtDNA occurs in the close vicinity of the mitoribosome (Zorkau et al., 2021). The mitochondrial membrane protein insertion routes seem to diversify from others like the bacterial and the endoplasmic reticulum insertion pathways, which are largely co-translational (Hegde and Keenan, 2021). In the following sections of this review we will first detail the structural features of the translocon of the OM (the TOM complex), which is the main entry gate for all mitochondrial proteins, and we will then discuss the mechanism and structural basis for the import of proteins into the OM and IM of mitochondria.

## The Translocon of the Outer Membrane Complex: The Main Gateway for Proteins to Cross the Outer Membrane

Nearly all mitochondrial membrane proteins are encoded in the nuclear genome and are synthesised by cytosolic ribosomes (Schmidt et al., 2010). The outer membrane therefore represents a significant barrier for these proteins which they must cross in order to be correctly assembled into both the inner and outer mitochondrial membranes. The entry gate that controls this import process is known as the TOM complex and is composed of a *β*-barrel pore forming protein (Tom40), a number of accessory/scaffolding proteins (Tom5, Tom6 and Tom7) and two receptor proteins (Tom20 and Tom70) that recognise mitochondrial targeting signals within protein sequences (Bolliger et al., 1995; Dietmeier et al., 1997; Rapaport and Neupert, 1999; Model et al., 2001; Gabriel et al., 2003; Mokranjac and Neupert, 2015; Pfanner et al., 2019; Wang et al., 2021). Additionally, the TOM complex contains a protein called Tom22, which appears to act as both a receptor and a scaffolding protein helping to control the number of pore-forming subunits associated to each fully assembled TOM complex as well as facilitating protein import (Lithgow et al., 1994; Bolliger et al., 1995; Moczko et al., 1997; Yano et al., 2000).

**FIGURE 2:**
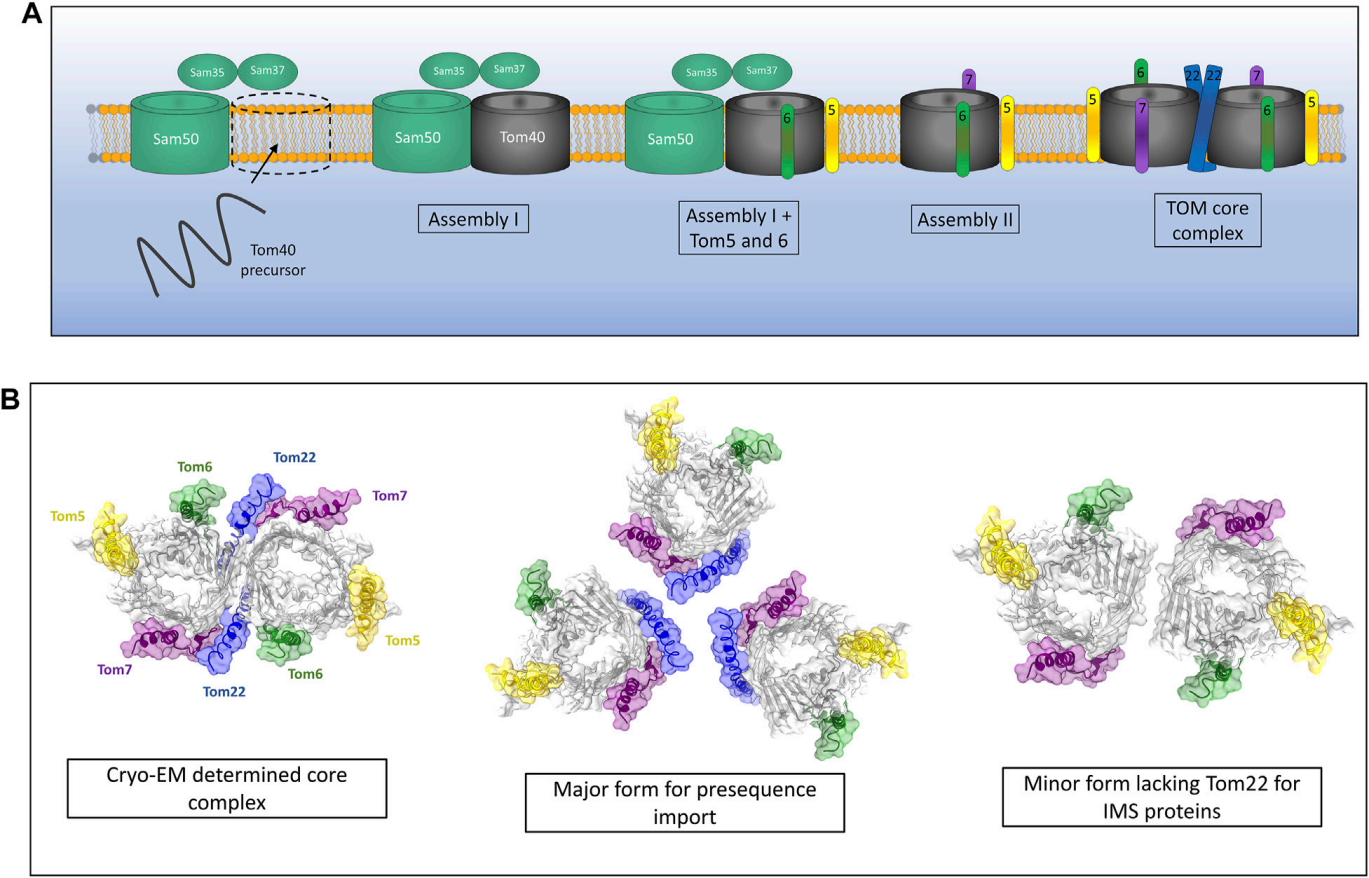
Assembly of the TOM complex. **(A)** The TOM complex is assembled sequentially in the outer membrane. First, a Tom40 precursor from the IMS is assembled into the outer membrane by the SAM complex (Assembly I). Subsequently, the small TOMs Tom5 and Tom6 are assembled with Tom40 while still associated with Sam50. The addition of Tom7 leads to dissociation of the TOM-SAM complex (Assembly II) allowing the final assembly of the TOM core complex *via* the introduction of Tom22. **(B)** Cryo-EM experiments identify a dimeric form of TOM containing Tom22. *In vivo* crosslinking data suggest that TOM actually exists as a trimer containing Tom22 and a dimer lacking Tom22 but containing Tom5, 6 and 7. The trimer makes up the majority of the population and is the predominant form for presequence import. The dimeric form lacking Tom22 facilitates the import of soluble IMS proteins. Models generated using PDB ID 6JNF.

The proteinaceous components of the TOM complex described above were identified and assigned many years ago (Kiebler et al., 1990), however it is only with recent advances in techniques such as cryo-EM that the assembly pathway of the TOM complex has become clearer. The assembly of the TOM complex is separated into three distinct stages known as assembly I, assembly II and TOM core assembly (Wang et al., 2021) (Figure 2A). Assembly I involves the integration of the Tom40 *β*-barrel protein into the outer membrane from the intermembrane space (IMS) side of the outer membrane *via* the sorting and assembly machinery (SAM) complex (this process will be discussed in detail later) (Wiedemann et al., 2003). This initial assembly stage requires a fully assembled and functional TOM complex for the initial translocation of Tom40 precursors across the outer membrane. Tom5 and Tom6 are subsequently inserted and assembled with Tom40 while it is still associated with the SAM complex (Dietmeier et al., 1997; Model et al., 2001; Becker et al., 2010; Thornton et al., 2010). The subsequent addition of Tom7 leads to dissociation of the growing TOM complex from SAM and constitutes assembly II (Yamano et al., 2010; Becker et al., 2011b; Wang et al., 2021). The addition of Tom22 to the complex leads to the assembly of the TOM core complex containing multiple fully assembled TOM complexes with Tom22 acting as a scaffold holding them together (Araiso et al., 2019; Tucker and Park, 2019; Wang et al., 2020b; Wang et al., 2021).

While the cryo-EM structures of the TOM core complex are an invaluable source of molecular detail and give us many clues as to the function of TOM as a protein import gate, they invariably show a dimer containing two Tom40 pores and two copies of Tom22 (Araiso et al., 2019; Tucker and Park, 2019). The multimeric state of the TOM complex in intact mitochondria seems to differ from these cryo-EM snapshots. Crosslinking studies have shown that the TOM complex is likely to exist predominantly as a trimer containing three *β*-barrel Tom40 subunits held together by three Tom22 subunits (Shiota et al., 2011). A minor dimeric form of the TOM complex was observed using cysteine-cysteine crosslinking and was found to contain two Tom40 *β*-barrels but no Tom22 (Shiota et al., 2015) (Figure 2B). It should be noted that both the trimeric and dimeric forms of the TOM complex contained the other accessory TOM components Tom5, Tom6 and Tom7. Interestingly, the trimeric form seems to be indispensable for import of pre-proteins into the mitochondria due to the presence of Tom20 and Tom22, both of which are required for presequence recognition and transport (Model et al., 2001). The dimeric form of Tom40 lacking Tom22 acts as an assembly intermediate allowing the dynamic exchange of new TOM subunits with the trimeric complex containing older subunits (Shiota et al., 2015; Araiso et al., 2021). Alongside this role, dimeric Tom40 can import several soluble MIA40 substrates into the IMS of mitochondria, specifically Tim9 and Cox17 (Gornicka et al., 2014; Sakaue et al., 2019). Larger TOM assemblies have also been observed via crosslinking analyses (Tucker and Park, 2019), although the physiological relevance of these larger oligomeric complexes remains to be discovered.

## Outer Membrane Protein Biogenesis

Aside from the vital role of the TOM complex in protein import, the outer membrane of mitochondria is also essential for maintaining the ionic balance of the organelle through the essential metabolite channel Porin/VDAC (Young et al., 2007), while also providing sites of contact between the endoplasmic reticulum (ER) and the mitochondria through mitochondrial distribution and morphology protein 10 (Mdm10) (Kühlbrandt, 2015; Ellenrieder et al., 2016). Given the vital nature of these functions the biogenesis of outer membrane proteins is a tightly regulated process requiring further essential components of the outer membrane. Membrane proteins of the outer mitochondrial membrane can be split into two distinct classes, the *α*-helical and *β*-barrel proteins.

Alpha-helical proteins are inserted directly into the outer membrane from the cytosol *via* the mitochondrial import machinery (MIM) complex in most cases (Doan et al., 2020). MIM is an oligomeric complex composed of two membrane spanning alpha-helical proteins, Mim1 and Mim2, with Mim1 being the major constituent of the complex (Becker et al., 2008; Hulett et al., 2008; Popov-Čeleketić et al., 2008; Dimmer et al., 2012). Mim1 is able to form pores in planar lipid membranes, while co-reconstitution with Mim2 does not substantially affect pore formation but may allow the recognition of positively charged residues in precursor proteins (Krüger et al., 2017). Mim1 (also known as Tom13), was originally characterised as a TOM complex assembly factor (Waizenegger et al., 2005; Lueder and Lithgow, 2009; Becker et al., 2011a). Given that the TOM complex contains a number of single-pass transmembrane alpha-helical proteins (Tom5, Tom6, Tom20 and Tom70) this is not surprising. Since these initial studies on TOM complex assembly, MIM has been identified as a key regulator for the assembly of outer membrane proteins Ugo1 and Fzo1, multi-spanning proteins involved in mitochondrial fusion (Becker et al., 2011a; Papić et al., 2011; Dimmer et al., 2012); as well as the multi-spanning protein Ubx2 (Mårtensson et al., 2019). Ubx2 is a dually localised protein resident in both the ER membrane, where it functions in the ER-associated degradation (ERAD) pathway, and the outer mitochondrial membrane where it performs a similar quality control function by removing stalled precursors from the TOM complex (Mårtensson et al., 2019).

**FIGURE 3:**
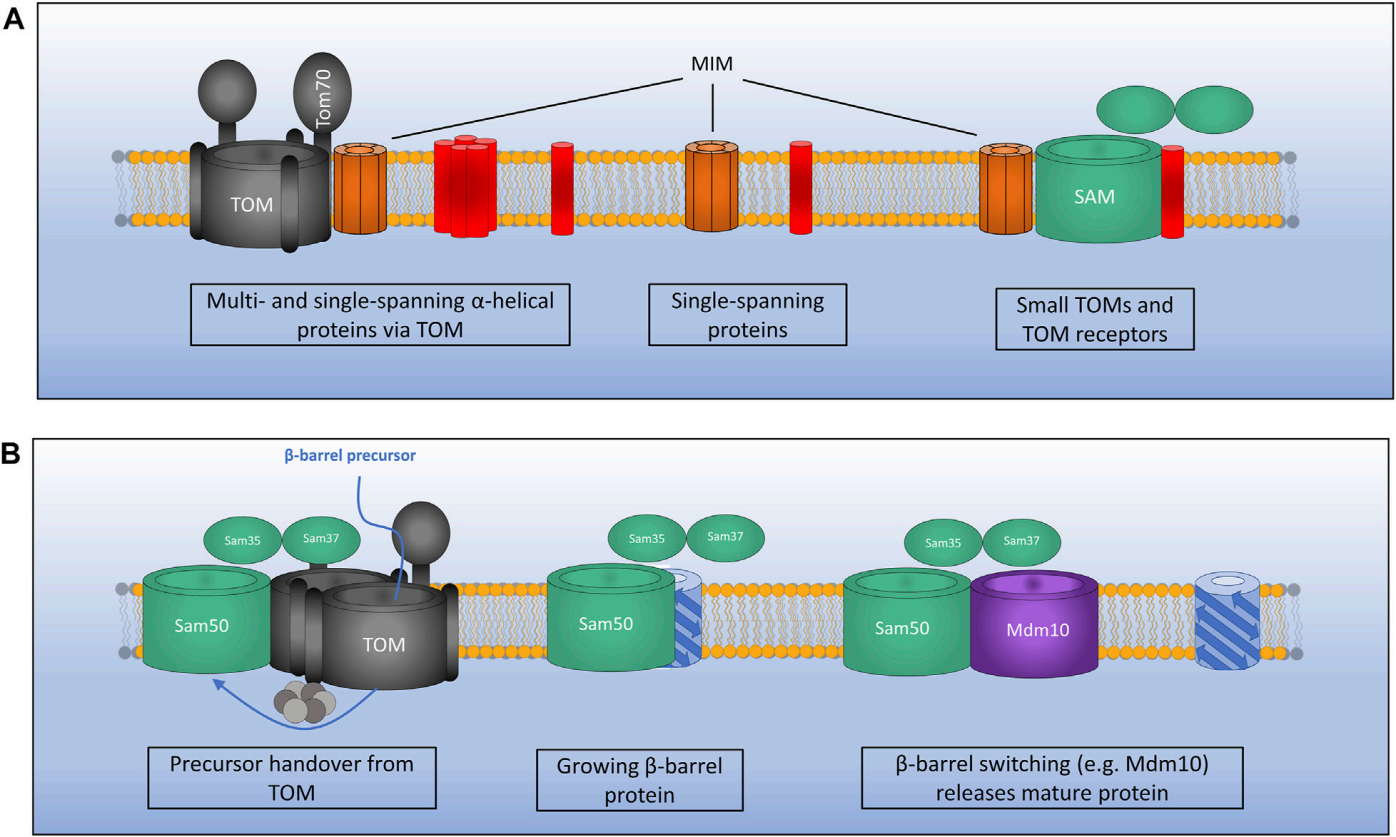
Mechanisms of outer membrane protein biogenesis. **(A)** The MIM complex exists in three distinct conformations: 1). In complex with TOM for the assembly of certain multi- and single-spanning *α*-helical membrane proteins which interact via the receptor Tom70.2). As a lone insertase for the assembly of certain single-spanning and tail anchored α-helical proteins. 3). In complex with SAM where MIM functions during assembly of TOM by inserting small TOM components into the growing structure. **(B)** The SAM complex inserts *β*-barrel outer membrane proteins *via* the IMS. Precursors pass through the TOM which is bound to SAM *via* Sam37 and the small TIMs in the IMS. TOM is then displaced as a growing *β*-barrel protein emerges from SAM. A switching mechanism is then required whereby a mature *β*-barrel protein (e.g., Mdm10) displaces the newly formed *β*-barrel allowing it to be released from the SAM complex.

Two interesting examples have also been found for MIM inserting proteins from the IMS as well as the cytosol (Song et al., 2014; Wenz et al., 2014). Outer membrane proteins Mcp3 and OM45 have both been identified as substrates of the MIM assembly pathway. What is interesting however is that initially these proteins are imported into the IMS via the presequence pathway involving the TOM complex. Prior to their insertion at the outer membrane by MIM they have also been found to interact with the TIM23 complex (Song et al., 2014; Wenz et al., 2014). This novel import route is interesting given the unknown functions of both of these proteins. A recent study has identified that the MIM complex exists in three distinct sub-populations (Figure 3A). As a lone insertase MIM acts on single spanning and tail anchored outer membrane proteins. MIM is also found in complex with the TOM and the SAM where it functions in multi-spanning outer membrane protein assembly and TOM assembly respectively (Doan et al., 2020). A protein complex performing the function of MIM has yet to be identified in mammalian cells, however a recent study was able to identify a functional equivalent to the MIM complex in trypanosomes (Vitali et al., 2018).

The proteins mentioned above represent a limited subset of outer membrane *α*-helical proteins and the insertion mechanism for many tail-anchored proteins remains to be properly elucidated. It has been suggested that some tail-anchored proteins are actually able to insert into the outer membrane without the assistance of any known insertase (Setoguchi et al., 2006; Kemper et al., 2008). This suggests that a spontaneous, thermodynamically driven mechanism may facilitate the insertion of these proteins, or there may be an as yet undiscovered pathway similar to the Get pathway of the ER (Asseck et al., 2021). It has also been postulated that the lipid composition of the outer membrane, specifically the presence of ergosterols, acts as a targeting factor for tail-anchored proteins (Krumpe et al., 2012).

The second class of outer membrane proteins are the *β*-barrel proteins, which form pores in the outer membrane for the transport of proteins and ions (Paschen et al., 2005). *β*-barrel precursors synthesised in the cytosol must first translocate across the outer membrane before being assembled from within the IMS by the SAM complex (Figure 3B) (Pfanner et al., 2004). Emerging precursors are stabilised in an unfolded state by the chaperones Hsp70 and Hsp40 proteins which recognise *β*-hairpins present in the precursors (Jores et al., 2018). Unfolded precursors are then trafficked to the mitochondrial outer membrane where they come into contact with the TOM complex *via* specific interactions between their *β*-hairpin structures and the receptor protein Tom20 (Jores et al., 2016). Unfolded proteins then pass through the outer membrane via the Tom40 channel, which is itself a *β* -barrel outer membrane protein required for its own import (Rapaport and Neupert, 1999).

As precursors emerge from Tom40 on the IMS side of the outer membrane they are able to interact with the small TIM proteins, IMS resident chaperones that protect the highly hydrophobic portions of membrane proteins from aggregation prior to correct assembly (Vial et al., 2002; Hoppins and Nargang, 2004; Wiedemann et al., 2004; Milenkovic et al., 2009; Weinhäupl et al., 2018).

Βeta-barrel precursors can then be assembled into the outer membrane by the SAM complex, an outer membrane protein complex consisting of a 16-stranded *β*-barrel protein Sam50 and two peripherally associated subunits Sam35 and Sam37 facing the cytosol (Kozjak et al., 2003; Milenkovic et al., 2004; Habib et al., 2005). Sam50 bears striking similarity to the bacterial outer membrane assembly protein BamA and indeed the assembly mechanism is also well conserved given that bacterial outer membrane proteins can be successfully imported and assembled into the mitochondrial outer membrane (Walther et al., 2009; Kozjak-Pavlovic et al., 2011; Ulrich et al., 2014).

Both Sam50 and Sam35 are essential proteins and have been found to interact with precursors through a motif known as the *β*-signal (Jores et al., 2016; Höhr et al., 2018). This motif (Polar-X-Gly-X-X-Hydrophobic-X-Hydrophobic) is present in the most C-terminal *β*-strand of precursor proteins and is required for membrane insertion. Sam50, being a *β*-barrel protein, also contains a *β*-signal in strand 16 (Imai et al., 2011). Recent crosslinking evidence suggests that an incoming *β*-signal is able to displace the endogenous Sam50 signal at a lateral opening in the Sam50 pore (Höhr et al., 2018). A *β*-barrel precursor associated with Sam50 through this *β*-signal interaction may then be able to grow and insert subsequent *β*-strands leading to a large assembly still associated with Sam50 in a similar manner as the recently proposed model for BAM insertion of *β*-barrel proteins in bacteria (Doyle and Bernstein, 2019). The role of Sam35 is yet to be fully elucidated, however some evidence indicates that Sam35 is required for protein insertion by Sam50 and interacts with the *β*-signal of precursors (Kutik et al., 2008). Sam35 is thought to be peripherally associated with the SAM complex facing the cytosol which is counterintuitive to a mechanistic understanding given that *β*-barrel proteins are inserted from the IMS side of the outer membrane. There is some evidence that Sam35 is actually embedded within the outer membrane through close interactions with Sam50 (Kutik et al., 2008), possibly within the pore of Sam50, although recent structural data do not support this hypothesis (Takeda et al., 2021; Wang et al., 2021).

The non-essential subunit Sam37 aids in the assembly of a SAM-TOM supercomplex through interactions with Sam35 and the cytosolic domain of Tom22 (Qiu et al., 2013; Wenz et al., 2015). These interactions, along with interactions with the small TIMs of the IMS, are thought to aid in precursor transfer from the TOM to the SAM during outer membrane protein biogenesis.

More recently, the outer membrane *β*-barrel mitochondrial distribution and morphology protein 10 (Mdm10) was identified as a transient component of the SAM complex important for the efficient assembly of the TOM complex (Meisinger et al., 2004, 2006). Mdm10 is dually localised to both the SAM complex and the ER mitochondria encounter structure (ERMES) which physically connects the mitochondrial outer membrane with the ER membrane and is thought to aid in lipid transfer between the two membranes (Kornmann et al., 2009; Flinner et al., 2013; Bohnert et al., 2015). Mdm10 acts as a membrane anchor for the rest of the ERMES complex subunits (Mdm34 and Mdm12) which connect to the ER through interactions with the ER protein Mmm1 (Kornmann and Walter, 2010).

The Mdm10 interaction with the SAM complex is mediated through Sam37 but also interestingly through Tom7, one of the small *α*-helical components of the TOM complex. Tom7 has an inhibitory effect on TOM complex assembly due to this dual interaction with both the TOM complex and Mdm10. Tom7 is able to interact with free Mdm10 which in turn favours Mdm10-ERMES assembly (Meisinger et al., 2006; Yamano et al., 2010). This has the effect of limiting the amount of Mdm10 which is able to bind to the SAM complex thus inhibiting TOM complex assembly. Cryo-EM experiments have recently generated high resolution structures of the SAM complex from *M. thermophila* and *S. cerevisiae* in complex with various substrates providing more evidence to support the mechanisms of outer membrane protein insertion discussed above (Diederichs et al., 2020; Takeda et al., 2021; Wang et al., 2021). The structural data clearly show a lateral opening in Sam50 between the *β*-signal in strand 16 and strand 1. Interestingly two of these studies were able to identify the SAM complex in association with Mdm10 (Takeda et al., 2021) and Tom40 (Wang et al., 2021). These structures have led to the *β*-barrel switching hypothesis which suggests that in order for a fully assembled *β*-barrel to be released by SAM into the outer membrane a dynamic switching event must take place with another *β*-barrel protein for this release to take place. It seems that for Tom40 this dynamic switching event is mediated by Mdm10 (Takeda et al., 2021; Wang et al., 2021), however for Porin this event seems to be mediated by a second monomer of Sam50 (Takeda et al., 2021). This SAM dimer complex will then dissociate to allow the start of the assembly process again. Further structural data are required to confirm this hypothesis however, as only fully assembled *β*-barrels have been visualised to date an assembly intermediate is missing. Such an intermediate has been observed in the bacterial BAM complex however which confirms the growth and release from the lateral opening (Tomasek et al., 2020). More work is also needed to understand SAM-mediated outer membrane assembly in humans given that yeast TOM cannot be assembled by human SAM (Wang et al., 2021).

## Protein Assembly by the Translocon of the Inner Membrane

**FIGURE 4:**
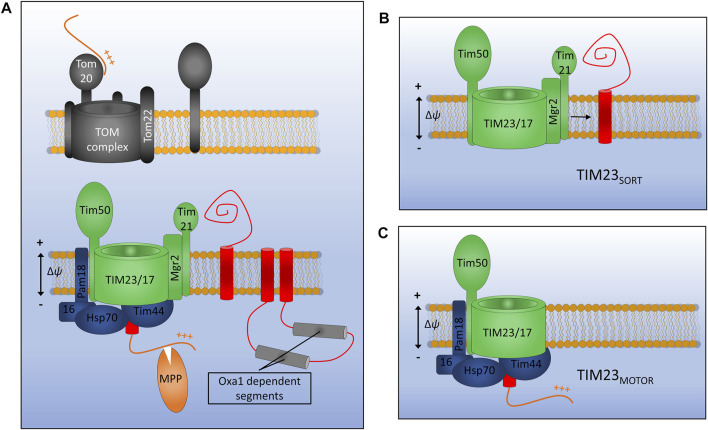
Assembly of inner membrane proteins by TIM23. **(A)** Presequence containing membrane proteins are recognised at the outer membrane by the Tom20 receptor and translocated across the outer membrane *via* Tom22 and Tom40. Once in the IMS presequences are recognised by the TIM23 subunit Tim50 and moved into the Tim23 channel. TIM23 can either laterally release stop-transfer segments into the membrane *via* the lateral gatekeeper Mgr2 associated with Tim21 **(B)**, or can translocate entire domains into the matrix via the PAM motor **(C)**. Motor function requires an inner membrane potential and ATP hydrolysis by mtHsp70. Once in the matrix the presequence will be cleaved by matrix processing peptidase (MPP). Certain multi-spanning proteins utilise both lateral diffusion directly from Tim23 and translocation for subsequent insertion *via* the Oxa1 insertase.

After passing through the TOM at the outer membrane, proteins containing a presequence that are destined for assembly at the inner membrane are transferred to the translocon of the inner membrane (TIM23) complex (Figure 4). TIM23 is a dynamic complex which adopts different conformations for the transport of proteins into the matrix and for partitioning certain membrane proteins into the inner membrane directly (Wiedemann and Pfanner, 2017).

Presequence containing precursors passing through the TOM complex will first interact with the receptor protein Tim50 which has a large receptor domain exposed to the IMS (Meinecke et al., 2006; Waegemann et al., 2015). In the absence of a bound precursor, Tim50 also acts as a plug for the Tim23 channel in order to maintain the permeability barrier of the inner membrane which is so crucial for the generation and maintenance of the proton gradient (Meinecke et al., 2006). Tim50 acts in concert with both the regulatory subunit Tim21 and the main channel forming polytopic membrane protein Tim23 to transfer precursors across the inner membrane and partition certain hydrophobic proteins into the inner membrane. Another membrane embedded subunit Tim17, which is a paralog of Tim23, is involved in maintenance of complex stability (Demishtein-Zohary et al., 2015).

Distinct conformations of the TIM23 complex have been identified that aid either the translocation of large hydrophilic domains across the inner membrane to the matrix, or the lateral diffusion of transmembrane *α*-helices into the lipid bilayer (Schendzielorz et al., 2018; Edwards et al., 2021). Key to these differing conformations are the TIM23 accessory proteins Pam18 and Mgr2. When Pam18 is bound to TIM23 (designated TIM23_MOTOR_), as is the case during precursor translocation, the lateral release of membrane proteins is inhibited. When TIM23 is associated with the subunits Tim21 and Mgr2 (designated TIM23_SORT_) hydrophobic protein sequences can be partitioned into the inner membrane in a process known as stop transfer (Chacinska et al., 2005, 2010; van der Laan et al., 2007; Bohnert et al., 2010; Schendzielorz et al., 2018). Mgr2 seems to act as a gatekeeper for protein lateral release due to its close association with the predicted lateral gate of Tim23 and its quality control-type effect on the lateral release process (Ieva et al., 2014; Matta et al., 2020). Following translocation or lateral release into the inner membrane, the presequence, which will have invariably been translocated into the matrix, is cleaved by the matrix processing peptidase (MPP). Interestingly, the TIM23 complex of yeast has been shown to interact with respiratory chain complexes in both the TIM23_SORT_ and TIM23_MOTOR_ conformations, indicating that a physical interaction keeping the translocase close to the site of proton motive force generation may be essential to the function of TIM23 in both the translocation and release of IM proteins and the translocation of soluble matrix proteins (van der Laan et al., 2006; Wiedemann et al., 2007).

In humans the TIM23 subunit TIM21 plays a distinct and important role in respiratory chain biogenesis. TIM21 was discovered as a component of an early cytochrome C oxidase assembly intermediate known as mitochondrial translation regulation assembly intermediate of cytochrome c oxidase (MITRAC) complex (Mick et al., 2012; Wang et al., 2020a). Knockdown of TIM21 also lead to complex IV assembly defects, while overexpression of TIM21 relieved ATP synthase assembly defects in yeast and improved the viability of human cell lines generated from patients with ATP synthase defects (Aiyar et al., 2014).

In order for TIM23 to transfer large protein domains into the matrix two things are essential: the mitochondrial membrane potential (Δ*ψ*) and the ATP-driven import motor PAM (Li et al., 2004; Van Der Laan et al., 2013). The major component of the import motor is the ATP-driven chaperone mitochondrial heat shock protein 70 (mtHsp70) which is connected to Tim23 *via* the peripheral subunit Tim44 which also aids in precursor transfer from Tim23 to mtHsp70 (Hutu et al., 2008; Banerjee et al., 2015). The co-chaperones Pam18 and Pam16 enable ATP hydrolysis by mtHsp70 while the nucleotide exchange factor Mge1 promotes the exchange of ADP for ATP and thus the recycling of the motor (Miao et al., 1997; Sakuragi et al., 1999; Wiedemann and Pfanner, 2017). The exact mechanistic details of how the motor operates and imports proteins into the matrix remains to be elucidated. Interestingly, a direct physical link has been found between Pam16 and Pam18 and the respiratory complex III-IV supercomplex (Wiedemann et al., 2007). This interaction is thought to facilitate the assembly of the PAM motor and may provide a key energetic environment to enhance protein translocation to the matrix.

The two distinct Tim23 pathways discussed above are most often used independently, however there is an example of a protein that utilises both mechanisms for its assembly in the inner membrane. Mdl1, a six transmembrane segment member of the ABC transporter superfamily, utilises both lateral diffusion and motor-driven translocation prior to its final assembly at the inner membrane (Bohnert et al., 2010). This study identified that of the six transmembrane helices of Mdl1, the first and last two diffuse directly into the inner membrane from TIM23_SORT_ while the two helices located in the middle of the protein sequence are fully translocated into the matrix by TIM23_MOTOR_ before being assembled into the inner membrane by the oxidase assembly protein 1 (Oxa1, whose function will be described later). Since this initial discovery further examples of proteins utilising a combination of stop-transfer and conservative (PAM-driven) mechanisms for assembly have been identified, for example Sdh4 (Park et al., 2013) and the Tim18-Sdh3 module of the TIM22 translocon (Stiller et al., 2016).

A high-resolution structure of the TIM23 complex is yet to be reported although given the speed of advancement of cryo-EM techniques a molecular structure will likely be available in the near future.

## Mitochondrial Carrier Proteins and the TIM22 Complex

**FIGURE 5:**
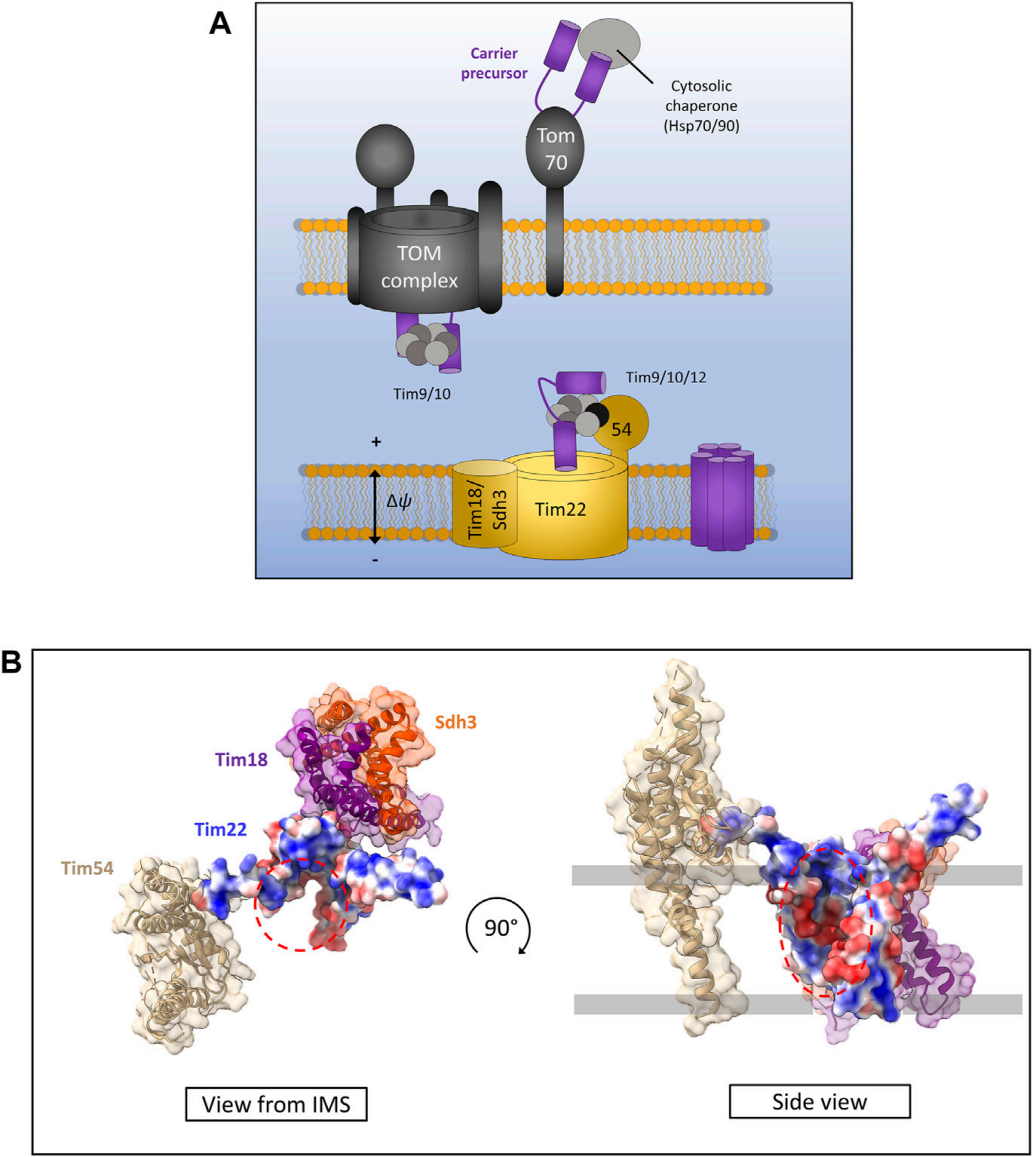
The carrier pathway for insertion at the inner membrane by TIM22. **(A)** Carrier precursors are recognised by cytosolic chaperones which move the precursor to the mitochondrial outer membrane via interactions with the receptor Tom70. Precursors are then translocated *via* the TOM to the IMS where they are met by the Tim9-Tim10 complex which maintains the hydrophobic precursor in an insertion-competent state. The precursor is then handed to the TIM22 complex via the membrane bound Tim9-Tim10-Tim12 complex which is bound to Tim22 via Tim54. Tim22 then inserts α-helical modules into the inner membrane in a membrane potential dependent manner. The Tim18/Sdh3 module associated with Tim22 is required to maintain complex stability. **(B)** Yeast Tim22 appears to contain a lateral gate exposing the hydrophilic core of the channel to the surrounding lipid suggesting a method of lateral diffusion directly from the channel. Lateral gate highlighted by red dashed areas. Models generated using PDB ID 6LO8.

The mitochondrial carrier proteins are a superfamily of 6 transmembrane *α*-helical proteins localised to the inner membrane of mitochondria (Figure 5A) and are essential for the transport of essential metabolites into and out of the mitochondrial matrix (Horten et al., 2020). The carrier proteins are synthesised without N-terminal presequences, and instead have internal targeting signals which can be recognised by the Tom70 receptor at the cytosolic face of the outer membrane (Sirrenberg et al., 1998; Diekert et al., 1999; Wiedemann et al., 2001; Kreimendahl et al., 2020). However, recent evidence suggests that Tom70 acts more like a recruitment factor for cytosolic chaperones such as Hsp70 and Hsp90 which are able to maintain the highly hydrophobic carrier proteins in an import-competent state and avoid unwanted aggregation (Backes et al., 2021).

Following translocation across the outer membrane through the Tom40 pore, carrier precursors interact with the small TIM chaperones in the IMS. Specifically, they bind the Tim9-Tim10 (Tim9 and Tim10a in humans) complex first at the IMS side of TOM (Luciano et al., 2001; Truscott et al., 2002; Vial et al., 2002) which forms a ring or doughnut-like structure that shields the hydrophobic domains of carrier substrates from the aqueous medium of the IMS (Weinhäupl et al., 2018). From here carrier precursors are handed to a second small TIM complex associated with the TIM22 translocon containing Tim9-Tim10-Tim12 in yeast and Tim9-Tim10a-Tim10b in humans (Sirrenberg et al., 1998; Lionaki et al., 2008; Qi et al., 2021). Interestingly, the outer membrane metabolite channel porin was recently identified to have a role in carrier protein biogenesis and bound directly to carrier protein precursors as well as directly recruiting TIM22 (Ellenrieder et al., 2019). The exact mechanism by which porin aids carrier insertion remains unknown but may involve physically linking the inner and outer membranes through these protein-protein interactions given that a direct TOM-TIM22 supercomplex does not appear to exist (Edwards and Tokatlidis, 2019; Ellenrieder et al., 2019; Horten et al., 2020).

Following outer membrane translocation and passage through the IMS chaperoned by the small TIMs, carrier precursors arrive at the TIM22 complex for their final insertion and assembly in the inner membrane. The TIM22 complex is composed of the main translocon protein Tim22 (Sirrenberg et al., 1996; Bauer et al., 1999) and a number of accessory proteins that are starkly different in yeast and humans. In yeast, the accessory proteins are Tim54, Sdh3 (which is also part of complex II), and Tim18. Tim54 contains a large IMS exposed domain which acts as a recruitment site for the Tim9-Tim10-Tim12 complex (Rehling et al., 2003, 2004). Tim18 and Sdh3 form a membrane integral module which is involved in the assembly of the TIM22 complex and is dependent on Oxa1 (Kerscher et al., 2000; Koehler et al., 2000; Gebert et al., 2011). In humans the accessory proteins are Tim29 and acylglycerol kinase (AGK) (Qi et al., 2021). Tim29 performs a similar function to yeast Tim54. It contains an IMS facing domain and is involved in interactions with the small TIMs and TIM22 complex assembly (Kang et al., 2016). AGK was only identified as a TIM22 complex subunit recently. Its role in carrier protein assembly is independent of its equally crucial role as a lipid kinase, however how it aids carrier protein biogenesis is not yet known (Kang et al., 2017; Vukotic et al., 2017).

The core component of the TIM22 complex in both yeast and humans is the translocase protein Tim22. The mitochondrial membrane potential is essential for precursor transfer from the small TIMs to Tim22 where carriers are laterally released as consecutive *α*-helical hairpin pairs into the inner membrane and adopt their functional fold (Wiedemann et al., 2001; Rehling et al., 2003). The mechanism of assembly and lateral release by Tim22 remains unknown, however recent structural analysis of both the human and yeast TIM22 complexes seems to indicate a cavity within Tim22 exposed to the lipid bilayer (Figure 5B) (Qi et al., 2021; Zhang et al., 2021). However, further structural data with bound precursors undergoing insertion are required to fully elucidate the carrier insertion mechanism.

As mentioned above, carrier proteins contain three modules each containing hairpin *α*-helical structures which are required for assembly by TIM22 (Wiedemann et al., 2001). Recently, a number of unconventional TIM22 substrates have been identified containing odd numbers of transmembrane helices in both yeast (Gomkale et al., 2020; Rampelt et al., 2020) and human (Acoba et al., 2021; Jackson et al., 2021) mitochondria. In yeast, the mitochondrial pyruvate carrier (MPC) proteins Mpc2 and Mpc3, both of which are predicted to have odd numbers of transmembrane *α*-helices (Bender et al., 2015), show a dependence on TIM22 for their assembly (Gomkale et al., 2020; Rampelt et al., 2020). TIM22 is also required for the assembly of MPC proteins in human cells (Gomkale et al., 2020). Furthermore, human cells also require TIM22 for the correct assembly of a number of sideroflexin (SFXN) proteins. These proteins are predicted to contain odd numbers of transmembrane α-helices and are essential as amino acid transporters in mitochondria that in turn affect mitochondrial one-carbon metabolism and respiratory complex III integrity (Kory et al., 2018; Acoba et al., 2021; Jackson et al., 2021). Taken together these recent studies suggest that the substrate spectrum of the TIM22 complex is much wider than previously thought, and biogenesis by TIM22 does not necessarily require modules of α-helical hairpin structures as originally thought.

## OXA1 and Respiratory Chain Assembly

**FIGURE 6:**
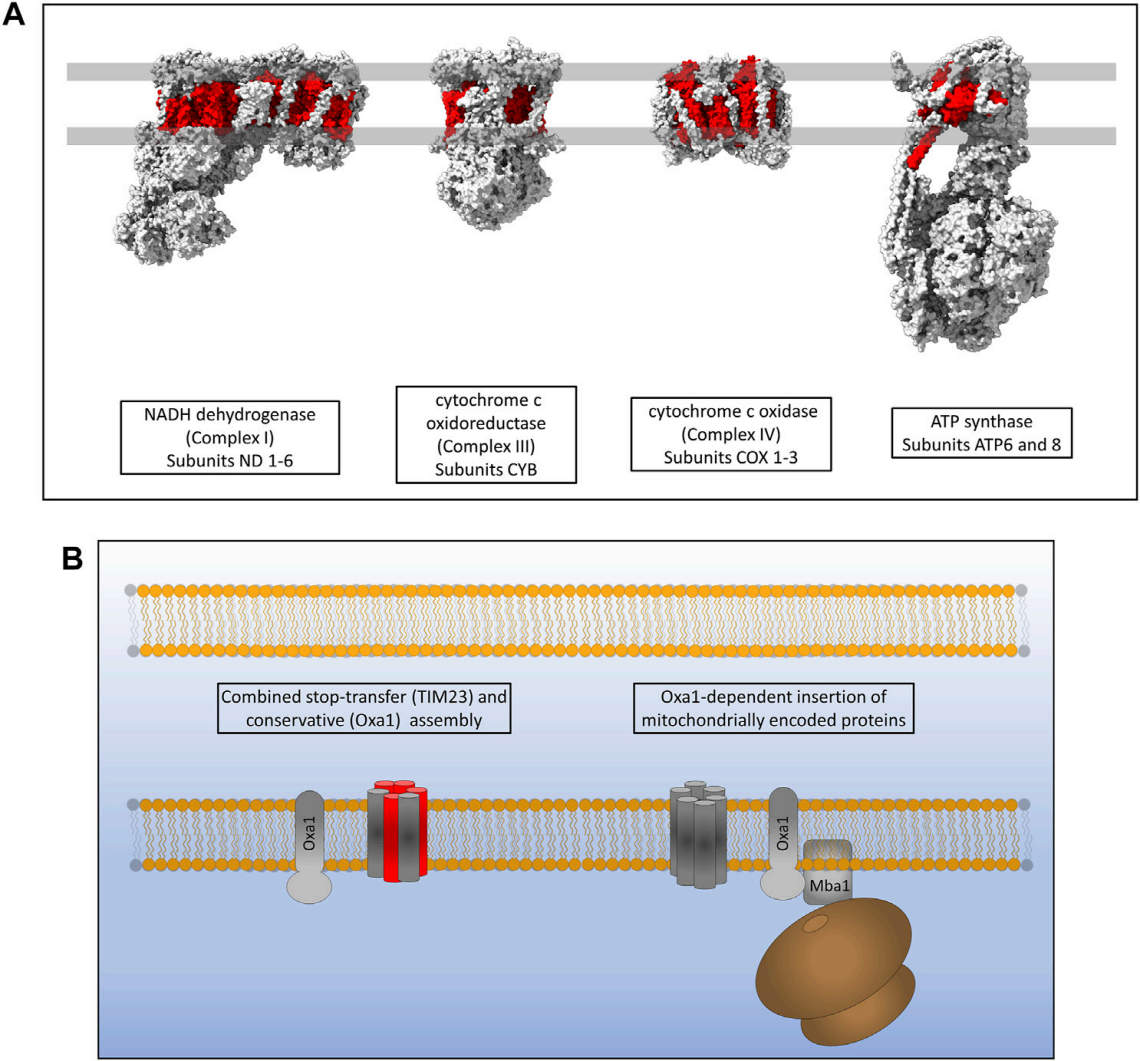
Inner membrane protein assembly by Oxa1. **(A)** Hydrophobic proteins of the human respiratory complexes that are synthesised on mitoribosomes are highlighted in red. **(B)** Oxa1 is able to insert proteins from two origins. Some proteins that are synthesised in the cytosol, passed through TOM and partially assembled by TIM23 require Oxa1 for the assembly of some of their transmembrane *α*-helices. Oxa1 also acts as the main, and only, insertase for mitochondrially encoded membrane proteins. The mitoribosome is physically associated with Oxa1 *via* Mba1 in yeast (mL45 in humans) whereby respiratory subunits are inserted and assembled into the inner membrane co-translationally.

Mitochondria maintain fully functional transcription and translation machineries, however many of the proteins making up these systems are encoded by nuclear DNA. The mitochondrial DNA (mtDNA) of eukaryotes codes for a small subset of highly hydrophobic membrane proteins that are subunits of the complexes of the oxidative phosphorylation (OXPHOS) respiratory electron transport chain (Taanman, 1999). Yeast mitochondrial genomes encode for 30–40 genes that include ribosomal RNAs, tRNAs and subunits of the OXPHOS machinery; specifically three subunits of ATP synthase (atp6, atp8 and atp9), three subunits of complex IV (cox1, cox2 and cox3) and a single subunit of Complex III (cytb) (Freel et al., 2015). Human mitochondria encode a total of 37 genes including, like yeast, ribosomal RNAs, tRNAs and OXPHOS subunits. Human mtDNA encodes 13 protein subunits of the OXPHOS machinery, two subunits of ATP synthase (atp6 and atp8), three subunits of complex IV (cox1, cox2 and cox3), one subunit of complex III (cytb) and 7 subunits of complex I which is not present in yeast (nd1, nd2, nd3, nd4L, nd4, nd5 and nd6) (Figure 6A) (Chocron et al., 2019). The inner mitochondrial membrane protein Oxa1 is essential for the correct insertion and assembly of many of these proteins and is therefore required for oxidative phosphorylation and cell viability (Figure 6B) (Thompson et al., 2018). Oxa1 was first identified in yeast and shares structural and functional homology with the bacterial insertase YidC (Bonnefoy et al., 1994; Altamura et al., 1996; Scotti et al., 2000). Like YidC, Oxa1 has a membrane spanning core of five *α*-helices that is absolutely essential for its insertase function (Kuhn et al., 2003; Hennon et al., 2015). The major difference between Oxa1 and YidC is the presence of a large C-terminal hydrophilic domain in Oxa1 located in the mitochondrial matrix. Removal of this C-terminal domain resulted in a loss of cell viability due to incomplete assembly of respiratory complexes, with the most drastic defects being present in complex IV (Szyrach et al., 2003). Furthermore, the C-terminal domain of Oxa1 was found to act as a binding site for the mitochondrial ribosome (Szyrach et al., 2003) suggesting that localising the translation of certain hydrophobic proteins to the membrane in close proximity to Oxa1 is key to their correct insertion and assembly. The membrane proximity of translation was also recently shown to be crucial for the thermodynamically driven assembly of a bacterial *α*-helical membrane protein in an entirely *in vitro* artificial system (Eaglesfield et al., 2021). The yeast protein Mba1 was also initially identified as a component of the respiratory chain assembly pathway acting independently of Oxa1 for the insertion of Cox2, cytochrome b and Cox1 (Preuss et al., 2001). Subsequent study of Mba1 identified it as a secondary mitoribosome receptor whose function seems to be both the anchoring of translation to the membrane through interactions with the mitoribosome and facilitating a tight interaction between Oxa1 and the mitoribosome (Ott et al., 2006; Keil et al., 2012). Knockdown of Oxa1 in humans results in a similar phenotype to yeast knockdowns, with the assembly of the respiratory chain being affected, specifically complexes I, IV and V (Stiburek et al., 2007; Thompson et al., 2018). Mba1 in humans is not freely associated with the membrane and Oxa1 as it is in yeast. It is instead a proteinaceous component of the human mitoribosome known as mL45 which still functions in attachment of the ribosome to Oxa1 when the mitoribosome is actively translating (Kummer et al., 2018; Itoh et al., 2021). Human Oxa1 also contains a long C-terminal hydrophilic domain which as a contact site for the mitoribosome (Itoh et al., 2021). Recent microscopic evidence has shown that the majority of translation in human mitochondria occurs at the cristae membrane close to the site of OXPHOS assembly by Oxa1 (Zorkau et al., 2021) and not at the nucleoid or RNA granules where mRNA is processed (Rey et al., 2020). Interestingly, the submitochondrial distribution of Oxa1 in yeast is altered depending on the energy demands of the cell. Under respiratory conditions Oxa1 is located mainly in the cristae due to the requirement for assembly of the OXPHOS machinery, however under fermentative conditions where OXPHOS is not generally required, Oxa1 is redistributed to the boundary membrane (Stoldt et al., 2012). An explanation for this may be that Oxa1 is also required for the assembly of Tim22 in the inner membrane and thus has a knock-on effect on the assembly of many carrier proteins which are still required under fermentative conditions (Hildenbeutel et al., 2012; Stiller et al., 2016).

While the function of Oxa1 as a membrane protein insertase is clear, a direct mechanistic understanding of its function remains elusive. It does appear to form voltage-gated ion channels when reconstituted in lipid membranes however the *in vivo* oligomeric structure as well as the mechanism allowing membrane protein lateral diffusion remain unclear (Krüger et al., 2012). No high-resolution structure for Oxa1 exists and would be a prerequisite to further our understanding of the role of Oxa1 in membrane protein biogenesis.

## Outlook

The field of mitochondrial membrane protein biogenesis has burgeoned in recent years. Advances in technologies such as cryo-EM have led to important discoveries enhancing our mechanistic understanding of this remarkably complex process. One thing that has become increasingly clear in recent years is the variety of membrane insertase and translocase complexes that exist within the mitochondria and appear to be highly adaptive, and most often act in concert with each other in dynamic ways to facilitate membrane protein biogenesis.

Even the relatively well understood presequence and carrier insertion pathways have revealed some new and exciting features recently. For example, the fact that the presequence (stop-transfer) pathway of TIM23 is able to work in conjunction with the Oxa1 insertase for the biogenesis of a number of inner membrane proteins, as well as the recent discovery of unconventional TIM22 substrates which suggests the recognition and insertion mechanism used by this insertase is more complex than previously thought.

A relatively unexplored avenue to date has been the involvement of the import and assembly protein Mia40 in oxidative folding required for membrane protein assembly. Mia40-dependent disulfide formation within transmembrane helices has however been identified in the essential proteins Tim22 and Tim17 (Wrobel et al., 2013, 2016). These disulfides may well stabilise transmembrane helical structure and could be important for other mitochondrial membrane proteins.

Despite all of these recent advances, there are still a number of key areas that require work to develop a full mechanistic understanding of the mitochondrial membrane protein biogenesis process. As discussed in this review, it is assumed that all of the known insertase complexes are able to laterally diffuse growing membrane proteins into the lipid environment through a “lateral-gate” type mechanism. While there is support for this theory based on the most recent structural data, what remains lacking for all of the mitochondrial insertases is a structural snapshot of this lateral diffusion process occurring. This has been shown for certain related bacterial insertases, but certainly an independent verification of this process by mitochondrial insertases would be incredibly valuable.

High-resolution structural data is still lacking for both the TIM23 complex and the Oxa1 insertase, while the recent structures of the TIM22 complex of both yeast and humans need further work to provide mechanistic details of insertion. It seems likely that the remarkable improvements in cryo-EM techniques will lead to structural data for these complexes being available in the near future and will likely provide us with further clues as to the insertion process. It will be very interesting to see structural snapshots of precursor protein translocation and biogenesis through all of the mitochondrial insertase complexes and to analyse these in conjunction with previously published cross-linking data to further elucidate the translocation and insertion process in mitochondria.
